# Validation of the French fear of missing out (FoMO) scale across the lifespan

**DOI:** 10.1007/s12144-025-08669-z

**Published:** 2026-01-17

**Authors:** Charikleia Lampraki, Daniela S. Jopp, Angélique Roquet, Julia Föcker, Marcelo de Maio Nascimento, Élvio R. Gouveia, Andreas Ihle

**Affiliations:** 1 Department of Psychology, University of Geneva, Geneva, Switzerland; 2 Center for the Interdisciplinary Study of Gerontology and Vulnerability, University of Geneva, Geneva, Switzerland; 3 Swiss Centre of Excellence in Life Course Research LIVES, Lausanne and Geneva, Switzerland; 4 Institute of Psychology, University of Lausanne, Lausanne, Switzerland; 5 Danish School of Education, Aarhus University, Copenhagen, Denmark; 6 College of Health and Social Science, University of Lincoln, Lincoln, UK; 7 Department of Physical Education, Federal University of Vale do São Francisco, Petrolina, Brazil; 8 Department of Physical Education and Sport, University of Madeira, Funchal, Portugal; 9 Laboratory of Robotics and Engineering Systems (LARSYS), Interactive Technologies, Lisbon, Portugal

**Keywords:** FoMO, Social media addiction, French validation, Lifespan, Measurement invariance

## Abstract

Since its development, the Fear of Missing Out (FoMO) scale has been widely used to measure addictive behaviour towards information and communication technologies. However, its French adaptation and associated psychometric properties have not been validated. This study aimed at addressing this gap in the literature and proposed a validated measure for French-speaking populations. Specifically, we investigated the factorial structure of the scale in French with a lifespan sample and test its age, sex, education, and time invariance. Data were derived from an online longitudinal study (2-waves, 2 months interval) with a lifespan sample (*N* = 543, age range18 to 98 years, *M* = 46.8). The results showed that the French adaptation had a different factorial structure than the original scale. Specifically, the selected model had excellent fit and identified three lower-order factors, based on 8 out of 10 original items, which were then loaded to one higher-order factor. Moreover, measurement invariance testing revealed that the scale can be used for mean level comparisons (scalar invariance) between men and women, high vs. low educated individuals and across measurement points, while caution is suggested when comparing young vs. older individuals (partial scalar invariance). Lastly, the findings supported convergent and discriminant validity of the scale. In conclusion, the French adaptation of the FoMO scale can be used in longitudinal studies and for mean comparisons across sexes, educational levels and time, while the factorial structure (three lower-order factors loading to one higher-order factor) can be used when modelling FoMO with structural equation methods.

**Supplementary Information:**

The online version contains supplementary material available at https://doi.org/10.1007/s12144-025-08669-z.

In recent years, the increasing use of technology and online communication tools has expanded opportunities for interaction and the maintenance of social networks across the lifespan (Baloran, 2020; Machin et al., 2022; Nowland et al., 2018). Such tools enable individuals, including those in rural areas, to strengthen ties with family and friends and engage in new social and leisure activities that promote healthy and active ageing (Astasio-Picado et al., 2022; Barbosa Neves et al., 2019; Wilson et al., 2023). At the same time, prolonged engagement with social media, online games and other digital platforms has raised concerns about addictive behaviours and their negative implications for physical, mental and cognitive health across the lifespan (Fancourt & Steptoe, 2019; Park et al., 2020; Wegmann et al., 2017). Against this backdrop, new psychological phenomena have emerged that warrant an in-depth and fine-grained investigation.

Within this framework, the Fear of Missing Out (FoMO) has emerged as a key psychological phenomenon linked to the rise of social media consumption. FoMO is commonly defined as the pervasive apprehension that others may be having rewarding experiences from which one is absent (Przybylski et al., 2013). Conceptually, it has been interpreted as consistent with processes described by social comparison theory (Festinger, 1957), as individuals evaluate their own experiences relative to those of others. At the same time, FoMO can also be understood through attachment theory, which emphasizes the fundamental human need for connectedness (Bowlby, 1969). First popularized in the media around 2010, FoMO has since attracted significant scholarly attention as it reflects concerns about exclusion from social events and experiences, often intensified by the visibility of others’ lives on social media (Andreassen et al., 2016; Elhai et al., 2020; Elmer et al., 2020; He et al., 2024; Milyavskaya et al., 2018). The need for connectedness is innate for humans at any stage of development (Bowlby, 1969). Social media and communication tools can facilitate connectedness with others without necessarily demanding excessive investment of time and resources for the development and maintenance of relationships, making them very attractive to users. At the same time, the perception of missing out on rewarding social experiences —and the fear of losing a sense of connectedness—has been shown to affect mental health. FoMO is most prevalent among adolescents and young adults, who are more proficient in using these technologies, with several studies indicating that a majority of university students report at least moderate levels (Elhai et al., 2020; Milyavskaya et al., 2018). However, with the increasing use of these tools also by older adults, FoMO may have important implications for mental health across the lifespan and specifically for old age.

To study this phenomenon, Przybylski et al. (2013) developed the FoMO scale, which has become the most widely used measure in its English version (Andreassen et al., 2016; Elhai et al., 2020; Elmer et al., 2020). The scale has been adapted into many languages including Arabic (Al-Menayes, 2016), Chinese (Li et al., 2021), Italian (Casale & Fioravanti, 2020), Korean (Joo et al., 2018), Portuguese (Almeida et al., 2024), Spanish (Martínez-Líbano et al., 2023), and Turkish (Gokler et al., 2016). However, findings regarding its structural validity have been inconsistent, with differences in the factor structure and the number of items included in the final version across languages. Recent studies also have investigated the validity of the FoMO scale in diverse contexts and communities (Durao et al., 2024; Joo et al., 2018; Wang et al., 2024), further contributing to our understanding of this phenomenon. We, therefore, selected the FoMO scale (Przybylski et al., 2013) for translation and validation in French because it is the most widely used instrument for assessing FoMO, enabling direct comparability with prior studies across diverse languages and cultural contexts.

Expanding the cross-cultural applicability of the FoMO scale is crucial, as cultural differences in communication styles, social norms, and technology use may shape how FoMO is perceived and expressed. Although French is one of the most widely spoken languages worldwide, a validated French version is, to the best of our knowledge, not currently available. This represents a significant limitation, as French-speaking populations span multiple continents (e.g., Europe, Africa, North America), where linguistic and cultural nuances may influence the experience and expression of FoMO.

In addition, important questions remain regarding the role of sociodemographic and temporal factors in shaping the experience of FoMO. Research to date has focused largely on adolescents and young adults (Elhai et al., 2025; Gul et al., 2022), yet with older adults increasingly engaging in digital communication, it is critical to examine whether the construct functions similarly across the lifespan. Previous findings on sex differences in FoMO have been inconsistent, with some studies reporting higher levels among women (Elhai et al., 2025) and others showing no clear pattern (Rozgonjuk et al., 2021), underscoring the need for further investigation. Education level may also be relevant, as it influences both access to technology and the ways individuals engage with social media, which could affect the salience of FoMO. Finally, testing measurement invariance across different assessment timepoints is crucial for longitudinal applications, ensuring that changes reflect true differences rather than shifts in measurement properties.

## The present study

Addressing these gaps, this study aims to translate and validate the FoMO scale in French, providing a reliable and culturally appropriate version for assessing this phenomenon in French-speaking populations. We also examine measurement invariance across key sociodemographic groups (age, sex, education), and two timepoints, in order to evaluate the stability and broader applicability of the measure. Finally, acknowledging prior evidence linking FoMO to social media use, personality, perceived stress, life satisfaction, and depressive symptoms (Elhai et al., 2020; Przybylski et al., 2013; Schneider, 2020; Stead & Bibby, 2017), we assess the convergent and discriminant validity of the French version with regard to these constructs.

## Methods

### Sample and procedure

The present study is based on a lifespan sample of 543 individuals, in which the age of the participants ranged from 18 to 98 years (*M* = 46.8). The sample size was determined based on recommendations from statistical literature suggesting at least 200 participants for factor analysis and 300 for confirmatory factor analysis (Thompson, 2004). The recruitment was performed using the snowball sampling technique. Specifically, study participants were Master students at the University of Lausanne and their family members, friends, and other acquaintances, recruited by them as a class assignment, leading to a diverse sample. Participants provided their informed consent, and they had to have reached adulthood (i.e., 18 years old and older), with females representing 63% (*n* = 347) of the sample, while 55% of the total sample reported being single. Regarding education, 44% (*n* = 237) had completed either a bachelor’s, master’s, or doctoral degree.

In terms of procedure, participants were informed about the aim of the study, the anonymity, confidentiality, and management of their data, as well as regarding the possibility to withdraw from the study at any time, before they gave their written consent to participate. The participation was voluntary, and the participants were not remunerated. We maintained data from participants that concluded both study waves, as incomplete data were considered as withdrawal from the study. The study was conducted online at two time points (2-month interval between waves; 12% attrition) and obtained the approval of the ethics committee of the University of Lausanne (C_SSP_092022_00002).

### Measures

#### Fear of Missing Out (FoMO)

We translated the English original 10-item scale from Przybyiski et al. (2013) into French. Following the instructions established for the English version, we asked participants to use a five-point Likert scale ranging from 1 = *not at all true of me* to 5 = *extremely true of me*. The items were: “I fear others have more rewarding experiences than me”, “I fear my friends have more rewarding experiences than me”, “I get worried when I find out my friends are having fun without me”, “I get anxious when I don’t know what my friends are up to”, “It is important that I understand my friends in jokes”, “Sometimes, I wonder if I spend too much time keeping up with what is going on”, “It bothers me when I miss an opportunity to meet up with friends”, “When I have a good time it is important for me to share the details online (e.g. updating status)”, “When I miss out on a planned get-together it bothers me”, and “When I go on vacation, I continue to keep tabs on what my friends are doing”. For the complete translated scale in French see Appendix A. Higher scores indicated higher FoMO.

#### Social media and technology use

Frequency of communication/social media and technology use was measured with the question “How often do you use a mobile device and/or laptop for…?”, separately regarding 13 different domains: emails, instant messaging, video calls, social networks, games, movies, reading the news, reading books, listen to music, taking pictures or videos, browsing the internet without specific reason, searching the internet for specific information, downloading applications or music, films etc. The answering format ranged from 0 = *never* to 4 = *everyday*. First, we calculated the mean composite score (average) across all 13 items listed above for an overall technology use score (Cronbach’s α = 0.91). Then, using only the four items addressing emails, instant messaging, video calls, and social networks we calculated a second mean composite score reflecting only communication and social media use (Cronbach’s α = 0.79). Higher scores indicated higher social media and technology use.

#### Personality

Neuroticism and extraversion were assessed with two items each from the BFI-10 (Rammstedt & John, 2007). Answering options ranged from 1 = *strongly disagree* to 5 = *strongly agree*. The average score was calculated for each dimension, and reliability was tested with the split-half calculation (Extraversion: *r* = .72; Neuroticism: *r* = .76). Higher scores indicated higher neuroticism or extraversion.

#### Short Perceived Stress Scale

The short 4-item Perceived Stress Scale (PSS-4) was used (Cohen et al., 1983). In addition, we also assessed item 3 from the longer original scale (i.e., In the last month how often have you felt anxious and “stressed”?) as a more direct measure of stress. Participants used a 5-point Likert scale to indicate the frequency of these emotions, ranging from 0 = *never* to 4 = *very often*. Negatively worded items were reversed to reflect higher stress. The scale composed of all 5 items had a high reliability (Cronbach’s α = 0.80). We computed a mean composite score with all 5 items, with higher values indicating more perceived stress.

#### Life Satisfaction

The 5-item Satisfaction with Life Scale (Diener et al., 1985) assessed how satisfied individuals were with their life. An example item is: “I am satisfied with my life”. A 7-point Likert scale was used ranging from 1 = *strongly disagree* to 7 = *strongly agree*. The scale had a high internal consistency (Cronbach’s α = 0.86). A mean composite score was calculated, using all 5 items, with higher values indicating higher life satisfaction.

#### Depressive symptoms

Depressive symptoms were measured with the 15-item CES-D scale (Center for Epidemiological Studies Depression Scale (Radloff, 1977). Participants were asked to indicate how often they experienced certain depressive symptoms in the past week (e.g., “I felt that I could not shake off the blues even with help from my family or friends”), using a 4-point Likert scale ranging from 1 = rarely/not at all (less than once a day) to 4 = most of the time/all the time (for 5 to 7 days). The scale had a high internal consistency (Cronbach’s α = 0.90). A mean composite score was calculated, using all 15 items, with higher values indicating more depressive symptoms.

#### Coding of demographic variables for subsequent analysis

For testing measurement invariance across different groups, we created various dichotomous variables based on demographic variables. More specifically, we created two age groups, with 0 = *less than 40 years old* and 1 = *40 years old and older* (47% of the sample). We coded sex as 0 = *men* and 1 = women (63% of the sample). We further created two education groups, with 0 = *low educated* and 1 = *highly educated individuals* (43%; completed bachelor’s degree or above).

### Analytical strategy

#### Preliminary analysis

First, we used zero-order correlations of the FoMO items to investigate the relationships between them and identify potentially redundant items at wave 1. Highly correlated items typically share a significant amount of variance and reduce the interpretability of the factor solution (Kyriazos & Poga, 2023), a problem commonly referred to as multicolinearity. Additionally, as Likert-type items rarely satisfy multivariate normality, we tested our data with Mardia’s test. Results indicated significant departures from normality (skewness = 1717.85, *p* < .001; kurtosis = 17.22, *p* < .001).

#### Exploratory factor analysis (EFA)

To investigate the underlying structure of the French version of the FoMO scale, we conducted an exploratory factor analysis (EFA). Factor extraction was guided by parallel analysis, a robust method for determining the number of factors, alongside inspection of the scree plot (Horn, 1965). We used principal axis factoring (PAF) as the extraction method, which performs well with non-normally distributed data, and an oblique rotation (Promax) given the likelihood of correlated factors.

#### Confirmatory factor analysis (CFA) and model comparison

Based on the EFA results and the proposed structure of the original measure (Przybylski et al., 2013), several models were tested and three are presented (for all tested models see Appendix D): (1) a single-factor with all the items, (2) a three-factor structure, and (3) a three-factor structure that composed a higher-order latent factor. The model fit was assessed using the Comparative Fit Index (CFI (Bentler, 1995)), the Tucker-Lewis Index (TLI (Tucker & Lewis, 1973)), the Root Mean Square Error of Approximation (RMSEA (Steiger, 1980)) and corresponding non-significant p-value at the 0.05 level, as well as the Standardized Root Mean Square Residual (SRMR (Bentler, 1995)), with thresholds of CFI/TLI ≥ 0.90, RMSEA ≤ 0.05, and SRMR ≤ 0.08 indicating good fit. We report χ² values for completeness, but given their sensitivity to sample size, model evaluation relied primarily on CFI, TLI, RMSEA, and SRMR. For model comparison, we relied on the Akaike Information Criterion (AIC (Akaike, 1974)), the Bayesian Information Criterion (BIC (Schwarz, 1978)), and the Sample Size-Adjusted BIC (SABIC (Sclove, 1987)) and log-likelihood values, with lower values indicating better fit.

#### Measurement invariance

We examined measurement invariance across age, sex, education, and assessment timepoints (study waves), to determine whether the model performed equivalently across different groups. Configural, metric, and scalar models were compared sequentially (Spini, 2003). Following current recommendations and given our sample size (*N* = 543), changes in CFI (ΔCFI ≤ 0.02) were used as the primary criterion. The CFI change is generally preferred over TLI change for this purpose, and it is less sensitive to sample size than χ² differences (Rutkowski & Svetina, 2014). An additional criterion used was the ΔRMSEA ≤ 0.015(Chen, 2007). When scalar invariance was not achieved, partial invariance models were considered to identify and address specific parameters causing non-invariance. First, modification indices were examined to identify items contributing to non-invariance. Partial scalar invariance was tested by freeing the intercepts of specific items based on empirical and theoretical justification (Putnick & Bornstein, 2016).

#### Convergent and discriminant validity

Finally, we examined associations between FoMO scores and relevant covariates, namely the mean composite score of frequency of social media and technology use, as well as personality (extraversion and neuroticism), perceived stress, life satisfaction, and depressive symptoms, based on prior research linking these constructs to FoMO. All analyses were conducted with R (Core Team, 2013), and the packages psych (Revelle & Revelle, 2015) and lavaan (Rosseel, 2012).

## Results

### Descriptive statistics and item correlations

Table 1 presents the mean and standard deviation for each FoMO item at wave one (for wave two see Appendix B). The highest mean value was for item nine (*M* = 2.94, *SD* = 1.32), while the lowest mean value was for item four (*M* = 1.30, *SD* = 0.74). The correlations between the study items showed either positive and significant (*r* = .11 – 0.79), or non-significant (only for items 8 and 9, *r* = .02 – 0.07) associations. The correlation matrix showed that items one and two correlated very strongly to each other with *r* = .79 (*r* = .83 for the same items in wave two), suggesting that the inclusion of both would result in redundant items in the factor analysis and misinterpretation of the model. Therefore, we decided to exclude item one (i.e., I fear others have more rewarding experiences than me) from the following factor analysis and maintain only item two (i.e., I fear my friends have more rewarding experiences than me).

**Table 1:** FoMO items: means, standard deviations, and correlations with confidence intervals in wave 1 (N =543)

	*M*	*SD*	1	2	3	4	5	6	7	8	9
1. I fear others have more rewarding experiences than me (Item 1)	2.10	1.21									
2. I fear my friends have more rewarding experiences than me (Item 2)	1.91	1.15	.79**								
			[.76, .82]								
3. I get worried when I find out my friends are having fun without me (Item 3)	1.71	1.06	.36**	.49**							
			[.28, .43]	[.42, .55]							
4. I get anxious when I don’t know what my friends are up to (Item 4)	1.30	0.74	.14**	.30**	.53**						
			[.06, .23]	[.22, .38]	[.47, .59]						
5. It is important that I understand my friends “in jokes” (Item 5)	2.58	1.29	.25**	.26**	.31**	.22**					
			[.16, .33]	[.18, .34]	[.23, .39]	[.13, .30]					
6. Sometimes, I wonder if I spend too much time keeping up with what is going on (Item 6)	1.94	1.18	.21**	.21**	.28**	.22**	.21**				
			[.13, .29]	[.13, .29]	[.20, .36]	[.14, .30]	[.12, .29]				
7. It bothers me when I miss an opportunity to meet up with friends (Item 7)	2.45	1.24	.15**	.18**	.35**	.29**	.26**	.36**			
			[.06, .23]	[.10, .26]	[.27, .42]	[.21, .36]	[.18, .34]	[.28, .43]			
8. When I have a good time it is important for me to share the details online (e.g. updating status; (Item 8)	1.42	0.78	.02	.04	.18**	.14**	.16**	.07	.18**		
			[-.07, .10]	[-.05, .12]	[.10, .26]	[.06, .22]	[.08, .24]	[-.02, .15]	[.10, .26]		
9. When I miss out on a planned get-together it bothers me (Item 9)	2.94	1.32	.08	.07	.18**	.12**	.11**	.28**	.51**	.11*	
			[-.01, .16]	[-.01, .16]	[.10, .27]	[.04, .21]	[.03, .20]	[.20, .36]	[.45, .57]	[.02, .19]	
10. When I go on vacation, I continue to keep tabs on what my friends are doing (Item 10)	2.22	1.28	.12**	.14**	.28**	.28**	.25**	.25**	.32**	.38**	.24**
			[.03, .20]	[.06, .23]	[.20, .36]	[.20, .36]	[.17, .33]	[.17, .33]	[.24, .40]	[.30, .45]	[.16, .32]

Values in square brackets indicate the 95% confidence interval for each correlation. * *p* < .05. ** *p* < .01

### Factor analysis

**Fig. 1:**
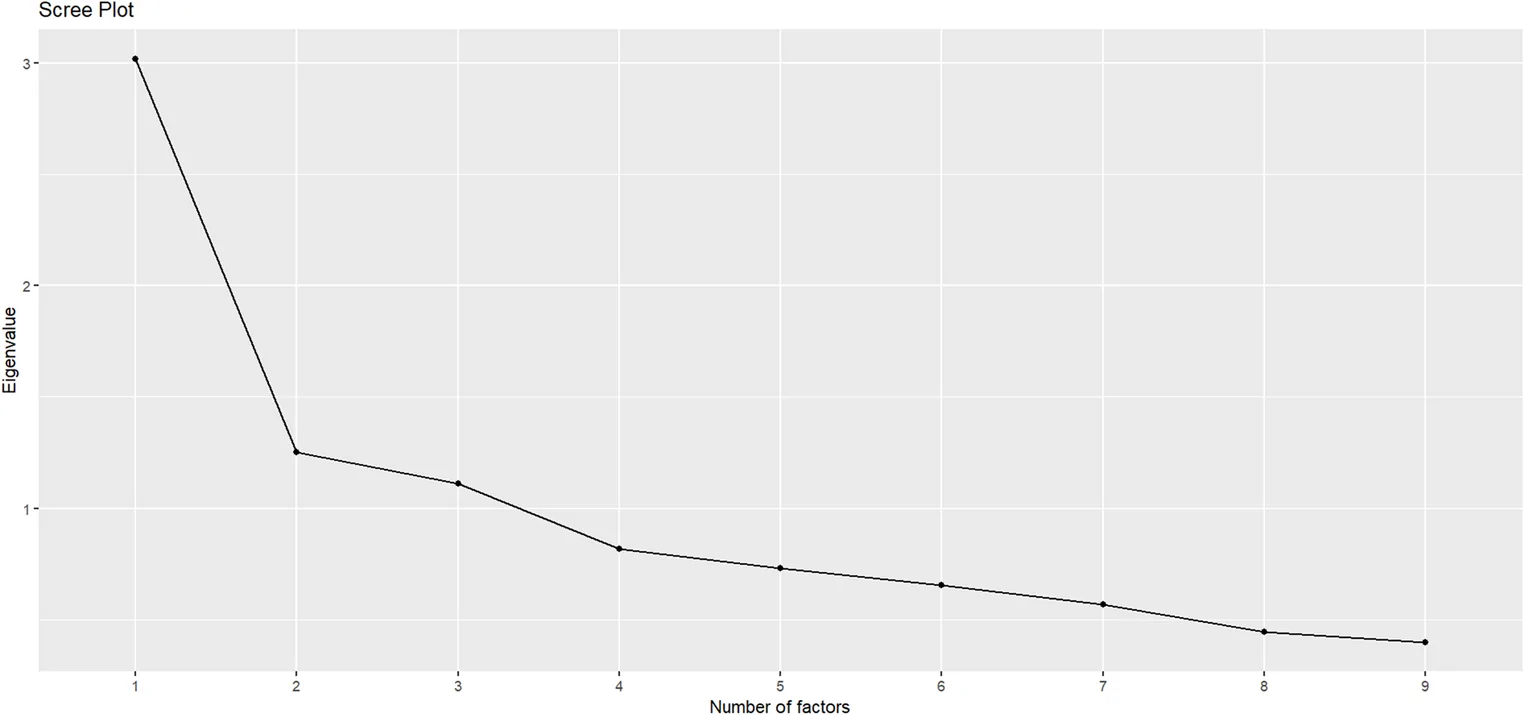
Scree plot of FoMO factors (exploratory factor analysis)

In order to examine whether the scale in French (for the exact translation see Appendix A) had, similarly to the English version, only one dimension, we first conducted an EFA with principal axis factoring, promax rotation. The value of the Kaiser-Mayer-Olkin Measure of Sampling Adequacy (KMO) was 0.77 suggesting that the size of the sample was adequate for conducting a factor analysis with nine items. Additionally, the Bartlet’s Test of Sphericity further supported the adequacy of the sample for the factor analysis with a significant chi-square value of 170.73 (*p* < .001). The EFA suggested three correlated factors (factor intercorrelation *r* = .39-0.42), with 3 items each loading on the first two factors and 2 items loading on the last one. The scree plot was inspected to identify the *elbow*—the point at which additional factors contributed diminishing incremental variance—also indicating a three-factor solution. Parallel analysis supported the retention of three factors: the first three observed eigenvalues (3.02, 1.25, 1.11) exceeded the corresponding simulated eigenvalues (0.37, 0.15, 0.11). Together with the scree plot and fit indices (SRMR = 0.02, RMSEA = 0.043, TLI = 0.96), these results indicated that a three-factor solution provided the best representation of the data. Item six (i.e., Sometimes, I wonder if I spend too much time keeping up with what is going on) exhibited weak loadings, ranging from 0.18 to 0.26, across two factors in EFA and was flagged for further evaluation and possible exclusion from subsequent models. Table 2 presents the factor loadings of the items for each factor (see Appendix c for the factor analysis figure). After extraction using principal axis factoring with promax rotation, the three retained factors explained 39% of the common variance (17%, 13%, and 9% respectively). Thus, the results were different from the original scale in English (Przybylski et al., 2013) which consisted of only one factor, although other validation studies, such as the Arabic and Chinese translations, also yielded more than one factors and supported the exclusion of 2 items from the final solutions (Al-Menayes, 2016; Li et al., 2021). The reliability of the two factors was acceptable with the first one having a Cronbach’s alpha level of 0.70 (0.72 for wave 2) and the second one of 0.65 (0.70 for wave 2). However, the third one had a split-half reliability of 0.55 (0.54 for wave 2) which was below the level of 0.60. Based on these results the first factor reflects anxiety of non-participation to friends-related experiences, the second one the importance of connecting with friends, and the third one the need for staying socially connected online (Fig. 1).

**Table 2:** Factor loadings of the exploratory factor analysis of the FoMO scale with promax rotation

	Factor 1	Factor 2	Factor 3
I fear my friends have more rewarding experiences than me (item 2)	**.68**		-.15
I get worried when I find out my friends are having fun without me (item 3)	**.87**		
I get anxious when I don’t know what my friends are up to (item 4)	**.58**		
It is important that I understand my friends “in jokes” (item 5)	.25	**.36**	
It bothers me when I miss an opportunity to meet up with friends (item 7)	-.18	**.73**	
When I miss out on a planned get-together it bothers me (item 9)		**.79**	
When I have a good time, it is important for me to share the details online (e.g. updating status; item 8)			**.66**
When I go on vacation, I continue to keep tabs on what my friends are doing (item 10)			**.62**
Sometimes, I wonder if I spend too much time keeping up with what is going on (item 6)	.25		.18
Eigenvalue	1.71	1.33	0.86
% of the variance explained	18.00	14.00	9.00
Cronbach's alpha	.70	.65	.55^a^

^a^For the third factor we used the split half reliability instead of the Cronbach’s alpha because we only had two items. The selected items for each factor are indicated with bold numbers. Items with loadings < 0.30 (e.g., for the item “Sometimes, I wonder if I spend too much time keeping up with what is going on”) were excluded from the factors’ structure

### Confirmatory factor analyses

Based on the results of the factor analysis and the solution proposed in the original scale (Przybylski et al., 2013), we conducted several confirmatory factor analyses (see Appendix D) to identify the best solution and validate the French version of the FoMO measure. The models presented in Table 3 are (1) the full item (ten) model with all items loading on a single factor (identical to the original version of the scale), (2) the proposed model from the factor analysis with 8 items loading on 3 factors, and (3) a model with the 8 items loading on 3 lower-order factors that load on one higher-order factor. The first model which forced all FoMO items (ten) to load on one factor yielded poor fit indices (e.g., CFI = 0.57 and TLI = 0.45 with a threshold of 0.95), indicating that a one factor solution (similar to the original scale) was not acceptable. We, then, tested the model proposed by the exploratory factor analysis presented above with three factors and 8 items. This model had an acceptable fit based on the fit indices (e.g., CFI = 0.97 and TLI = 0.95). However, as a third model, we tested three lower-order factors (same as in the second model) representing the sub-dimensions, while also specifying a higher-order factor, representing a combination of the previous two models. This higher-order factor model had similar model fit to the second model (e.g., CFI = 0.97 and TLI =0.95), but reflected better the original structure of the measure in English as proposed by the authors (Przybylski et al., 2013).

**Table 3:** Confirmatory Factor Analyses of FoMO Scale

	1-Factor	3-Factors			1 Superfactor with 3-Subfactors		
		Factor 1	Factor 2	Factor 3	Factor 1	Factor 2	Factor 3
I fear others have more rewarding experiences than me (item 1)	.61	-	-	-	-	-	-
I fear my friends have more rewarding experiences than me (item 2)	.69	.53			.53		
I get worried when I find out my friends are having fun without me (item 3)	.72	.89			.89		
I get anxious when I don’t know what my friends are up to (item 4)	.53	.61			.61		
It is important that I understand my friends “in jokes” (item 5)	.46		.46			.46	
Sometimes, I wonder if I spend too much time keeping up with what is going on (item 6)	.40	-	-	-	-	-	-
It bothers me when I miss an opportunity to meet up with friends (item 7)	.48		.60			.60	
When I miss out on a planned get-together it bothers me (item 8)	.31		.82			.82	
When I have a good time, it is important for me to share the details online (item 9)	.22			.45			.45
When I go on vacation, I continue to keep tabs on what my friends are doing (item 10)	.40			.84			.84
Superfactor loadings	-	-	-	-	.73	.64	.64
CFI	.56	.97			.97		
TLI	.43	.95			.95		
RMSEA	.19	.05			.05		
RMSEA p-value	<.001	.36			.36		
SRMR	.10	.04			.04		
Chi2	828.56	42.67			42.67		
Chi2 p-value	<.001	<.001			<.001		
Df	35	17			17		
AIC	13710.78	12207.15			12207.15		
BIC	13826.06	12323.17			12323.17		
SABIC	13740.36	12237.46			12237.46		

### Measurement invariance

Measurement invariance was based on the third confirmatory factor model with 3 lower-order factors and one higher-order factor (Table 4). We tested whether the model was consistent across age, sex, education, and timepoints (wave 1 vs. wave 2). Configural and metric invariance were supported across all groups. For sex, education, and timepoints, scalar invariance was also supported. However, scalar invariance was not achieved for age (young vs. old) due to poor fit (ΔCFI = 0.026, RMSEA = 0.066). After careful consideration we identified that the item “When I go on vacation, I continue to keep tabs on what my friends are doing” contributed to the lack of fit. Following the recommendations from Byrne and colleagues (Byrne et al., 1989), this item was removed (after also having tested allowing its intercept to vary), and the invariance testing process was repeated. After this adjustment, scalar invariance was achieved, with acceptable fit indices (e.g., CFI = 0.946, ΔCFI = 0.010, RMSEA = 0.059). These results indicate that meaningful latent mean comparisons can be made between young and older individuals for the revised model (i.e., two subfactors and a single item – previously part of the third subfactor – loading on one superfactor; see Table 4).

**Table 4:** Measurement Invariance of the Model with Three Subfactors and One Superfactor, Comparing Young vs Older Aged Individuals, Men vs Women, Higher vs Lower Educated Individuals, and Wave 1 vs Wave 2

	CFI	ΔCFI	TLI	RMSEA	RMSEA p-value	SRMR	Chi2	Chi2 p-value	df	AIC	BIC	SABIC
Age (young vs old)												
Configural Invariance	.969	-	.949	.050	.463	.044	59.464	<0.01	34	11934.84	12166.78	11995.37
Metric Invariance	.956	.013	.940	.054	.353	.054	74.223	<0.001	41	11938.76	12140.63	11991.44
Scalar Invariance (not achieved)	.930	.026	.913	.066	.095	.067	96.297	<0.001	45	11955.13	12139.82	12003.33
Partial Scalar Invariance with Intercept of Item 10 Freed (achieved)	.941	.015	.925	.061	.184	.061	87.256	<0.001	44	11947.87	12136.86	11997.19
Sex (men vs women)												
Configural Invariance	.953	-	.923	.065	.132	.048	71.989	<.001	34	12110.38	12342.02	12170.61
Metric Invariance	.956	-.003	.940	.057	.274	.053	74.472	<.001	41	12102.45	12304.07	12154.88
Scalar Invariance	.952	.004	.940	.057	.270	.056	81.154	<.001	45	12102.83	12287.29	12150.79
Education (higher vs lower)												
Configural Invariance	.972	-	.953	.051	.455	.044	58.190	.006	34	12154.30	12386.24	12214.82
Metric Invariance	.972	0	.962	.046	.599	.050	63.929	.012	41	12148.23	12350.11	12200.92
Scalar Invariance	.957	.015	.946	.054	.350	.055	79.417	.001	45	12157.64	12342.33	12205.84
Time (wave 1 vs wave 2)												
Configural Invariance	.960	-	.934	.066	.036	.043	105.691	<.001	34	22622.88	2289.34	22717.83
Metric Invariance	.959	.001	.944	.061	.095	.050	111.782	<.001	41	22619.99	22851.90	22702.63
Scalar Invariance	.958	.001	.948	.059	.133	.051	117.002	<.001	45	22619.00	22831.18	22694.61

The results, with the exception of age, thus supported a similar performance of the FoMO scale across the tested groups and measurement points. For age, the results supported a similar performance of the FoMO scale but only with the removal of the problematic item.

### Correlational analysis for convergent and discriminant validity

Correlational analysis revealed that FoMO was, as expected, strongly and positively related to more frequent overall technology use, and to online communication and social media use (*r* = .58, *p* < .001 and *r* = .56, *p* < .001, respectively). Regarding personality, FoMO was not related to extraversion (*r* = .07, *p* > .10) but it was moderately associated with higher levels of neuroticism (*r* = .43, *p* < .001). Higher levels of stress and depressive symptoms were moderately associated with higher levels of FoMO (*r* = .39, *p* < .001 and *r* =.46, *p* < .001, respectively). Lastly, life satisfaction was not significantly related to FoMO (*r* = -10, *p* > .10). These results provide evidence for the convergent validity of FoMO with technology use, social media use, neuroticism, stress, and depressive symptoms and discriminant validity with extraversion and life satisfaction, as expected.

## Discussion

The present study validated a French version of the FoMO scale (Przybylski et al., 2013) with a lifespan sample, and provided important information on the usefulness of the measure across different population subgroups. Fear of missing out on valuable moments has been related in the past with higher frequency of social media use, more stress and depressive symptoms, lower quality of life and satisfaction, contributing to the vulnerabilisation of affected individuals across the lifespan (Andreassen et al., 2016; Elhai et al., 2020; Elmer et al., 2020; Milyavskaya et al., 2018). However, it is necessary to assess FoMO with reliable and trustworthy instruments adapted to specific contexts and populations. This study makes three key contributions: it is the first to validate a French adaptation of the FoMO scale, provides evidence for its psychometric properties (factorial structure, reliability, invariance, validity), and offers insights into how FoMO should be modelled in complex analyses such as structural equation modelling.

First, it is important to mention that the modelling of the full 10 item French FoMO scale did not yield any acceptable results, and further investigation led to the exclusion of two items for the subsequent analyses. Factor analysis revealed not one single factor, as proposed by the original FoMO scale publication (Przybylski et al., 2013), but three underlying factors: One factor reflected the anxiety of not participating to friends-related experiences, a second factor focused on the importance of connecting with friends, and a third factor mirroring the need for staying connected to social media. Further analysis using confirmatory factor analysis revealed that the three underlying factors composed one higher-order factor, which reflected the single factor structure originally presented in Przybyiski et al. (2013) English FoMO scale. The internal consistency of the higher-order factor was acceptable, thus, our study provides support to the idea that FoMO can be represented as unidimensional construct, yet made up of underlying subdimensions.

These cross-cultural differences in the factor structure have been observed before in other language translations, suggesting that some of the items of the scale create subscales that may also differ depending on the language and population. Importantly, Al-Menayes (2016) and Martinez-Libano et al. (2023), who also tested the scale in Arabic and Spanish with exploratory factor analysis, found different factorial structures to the original one, with two underlying factors. Moreover, it is of note that, similar to this study, the Korean validation study (Joo et al., 2018) also found three underlying factors, with one consisting only of two items. However, only the Spanish (Martínez-Líbano et al., 2023) and Korean (Joo et al., 2018) studies performed a confirmatory factor analysis as we did. In conjunction, the findings suggest that although the scale can be used as a single, mean composite construct, it is possible to use its items separately if research questions focus on specific aspects of FoMO.

These variations observed across different language adaptations highlight the potential impact of cultural differences on how individuals interpret and experience FoMO. These differences may stem from variations in communication styles, social norms, or cultural values regarding friendships and social media use. For instance, collectivist cultures may emphasize group-related anxiety, while individualist cultures may place greater importance on personal connections or social media presence. In addition, differences in technology adoption and use across cultural contexts may shape how FoMO manifests: in some populations, social media is central to maintaining family ties, while in others it primarily reflects peer-oriented entertainment (Liu, 2025). Generational expectations and social roles may further interact with culture, meaning that FoMO in younger adults often centres on peer belonging, while in older adults it may reflect perceived exclusion from family or intergenerational activities. Such findings underscore the importance of considering cultural communication styles and norms when using the FoMO scale in cross-cultural research.

In addition, apart from the apparent advantage of this version for French-speaking populations, it is also more suitable for use in specific contexts when we opt for smaller questionnaires. Lastly, while future research aiming at investigating FoMO should confirm the underlying factor structure with structural equation modelling, such studies may also benefit from investigating FoMO considering the underlying subdimensions, as these may lead to more nuanced results and conclusions.

Another important contribution of this paper is the investigation of measurement invariance across age groups, sex, educational levels, and measurement points. Our results revealed that the FoMO scale performs similarly across subgroups and can be reliably used for comparisons between men and women, highly vs. low educated individuals as well as across measurement points. Our findings regarding sex replicate the results of Martinez-Libano et al. (2023) who tested measurement invariance (scalar) in FoMO in a sample of Chilean university students. Another important finding which, to our knowledge, has never been investigated in the past was establishing measurement invariance between highly vs. low educated individuals. The results show that mean differences detected in FoMO with regards to educational levels can thus be attributed to education itself and not to structural inconsistencies of the scale. Regarding age, we found partial scalar invariance, indicating that even though the factor structure is the same across age groups, when testing mean level comparisons between them one of the items was not group-invariant. The vacation-related item (“When I go on vacation, I continue to keep tabs on what my friends are doing”) did not function equivalently across younger and older adults. While statistical diagnostics (e.g., modification indices, intercept non-invariance) highlighted this misfit, there is also a conceptual explanation. The behaviours referenced in this item may carry different meanings across age groups: for younger adults, vacations are often intertwined with maintaining digital connectivity and sharing experiences online, whereas for older adults, vacations may be more strongly associated with disconnection from social media or different leisure routines. This differential interpretation likely contributes to its lack of scalar invariance. In line with recommendations to combine empirical and theoretical considerations in measurement evaluation, we therefore treated this item as non-invariant, suggesting that the scale had to be further adapted to allow for age-group mean level comparisons and to achieve partial scalar invariance. We note that the remaining items demonstrated consistent measurement properties, supporting the robustness of the revised FoMO scale across age groups. Essentially, these findings suggest that FoMO was developed for younger aged individuals and that the scale must be further adapted if the individuals assessed are substantially older in age.

Our findings also show that within our French lifespan sample, FoMO is associated to various behavioural and psychological aspects, including technology and social media use, perceived stress, personality and depressive symptoms, strengthening evidence provided by earlier studies (Elhai et al., 2020; Przybylski et al., 2013; Schneider, 2020; Stead & Bibby, 2017). These findings provide evidence of convergent validity for the French version of the FoMO scale: strong positive associations with constructs theoretically related to FoMO, such as technology use, social media use, neuroticism, stress, and depressive symptoms. More specifically, we found that individuals with higher FoMO were more likely to have a higher technology and, specifically, social media use, which is in line with prior work (Elhai et al., 2020; Przybylski et al., 2013). Our findings further suggest that individuals with higher FoMO were more likely to have higher neuroticism levels, which had also been demonstrated earlier (Stead & Bibby, 2017). In line with Stead and Bibby (2017), we found that extraversion was not related to FoMO.

Moreover, our findings demonstrated that individuals with high FoMO were at risk for poorer mental health, as they reported higher perceived stress and depressive symptoms, which had also been seen in prior studies (Elhai et al., 2020; Schneider, 2020). These associations point to the potential value of targeting FoMO in mental health interventions. For example, interventions addressing FoMO could incorporate cognitive-behavioral therapy to help individuals manage the stress and emotional difficulties associated with high levels of FoMO. Clinicians might also consider assessing FoMO as part of routine evaluations for individuals who exhibit signs of excessive social media use or technology-related anxiety. Given the link between FoMO and neuroticism, personality-focused approaches, such as resilience training or emotion regulation strategies, could be tailored to individuals who may be more vulnerable.

As FoMO reflects addictive behaviour to digital technology, our findings are in line with past literature indicating that high use may be harmful for younger ages but moderate use may be beneficial (Przybylski & Weinstein, 2017). However, we did not find an association between FoMO and life satisfaction or extraversion in contrast to past research (Przybylski et al., 2013; Stead & Bibby, 2017). These results provide support for the discriminant validity of the French version of the FoMO scale. A possible explanation may be that our sample covers the adult lifespan up to the age of 98 and age-specific association may not be captured. In addition to the partial scalar invariance results, proposing attention when comparing young to older aged groups, FoMO may be more important for the life satisfaction of younger individuals but not in middle-aged or older adults. Past research has documented that increased social media use, which may be reflected in high FoMO scores, is related to worse life satisfaction one year later in young adults and specifically in 19-year-olds (Orben et al., 2022). Indeed, follow-up analyses (not presented) indicated that life satisfaction in our sample related negatively and significantly only to younger adults. These findings point out that although FoMO becomes relevant for older adults due to their familiarization with information and communication technologies, the relationships and mechanisms found for the younger adults are not necessarily similar in old age. These findings underscore the importance of developing age-sensitive interventions. For younger adults, interventions could focus on reducing excessive social media use or encouraging more meaningful, offline social connections. For older adults, as FoMO may become more relevant with increased use of technology for social interactions, interventions could help support older individuals in navigating social media in ways that promote well-being without contributing to FoMO-related stress or dissatisfaction. Further investigation is needed to explore these relationships and mechanisms in older populations, especially as they become increasingly involved in social media and digital technology.

Although this study has several strengths, it also presents some limitations. First, the factorial structure of FoMO that we identified shows similarities but also differences from the original scale. Although we found a higher order factor that is similar to the scale developed by Przybylski et al. (2013) we had to exclude one item (i.e., I fear others have more rewarding experiences than me) because it was very strongly correlated with another item (i.e., I fear my friends have more rewarding experiences than me) and the factor analysis also proposed that one item (i.e., Sometimes, I wonder if I spend too much time keeping up with what is going on) did not load on any of the three factors. In addition, one limitation of the current validation is that one of the subdimensions—the “online connectedness” factor—was represented by only two items. The two items—“When I have a good time, it is important for me to share the details online” and “When I go on vacation, I continue to keep tabs on what my friends are doing”—reflect a distinct dimension of the construct (i.e., need for staying connected to social media) that was consistently identified across multiple analyses (i.e., exploratory and confirmatory factor analyses). This alignment suggests that, despite its brevity, the factor captures a meaningful and coherent construct, similar to the Korean version of the FoMO where one of the extracted subfactors consisted of only two items (Joo et al., 2018). While short subscales are often less reliable, our analyses support this distinct and theoretically meaningful aspect of FoMO, namely the tendency to remain socially connected and visible through digital platforms. Still, we acknowledge that two-item factors are more vulnerable to measurement non-invariance, which was reflected in our age analyses, where the “vacation” item functioned differently for younger and older adults. Moreover, factors with few items may lack internal consistency, as Cronbach’s alpha and similar measures tend to perform poorly when there are fewer than three items. However, in our study we used the split half reliability, which is suitable for two items and had an marginally acceptable value (.55), providing empirical justification for retaining this two-item factor in the current study. Finally, alternative approaches, such as adding additional items to strengthen the factor, were not feasible within the scope of this study. The excluded items of the translated version did not provide theoretical or empirical justification for including them in the factor. Future research may address this limitation by developing and validating new items that expand the conceptual scope of this dimension while ensuring psychometric robustness.

A possible explanation for the differences and similarities found in the structure of the French version of the FoMO scale may be that, although we offer new insights with a lifespan sample, covering up to 98 years of age, the original scale was developed and validated with a much younger sample, with the oldest individual being 62 years old. Moreover, younger aged individuals may have a more advanced level of digital proficiency compared to their older aged counterparts, resulting in more frequent use of information and communication tools, and, eventually, to a different experience of FoMO. Another possible explanation may be that the translated version of the two highly correlated items may measure the same aspect of FoMO, making the use of both redundant. Therefore, future research must replicate and expand these findings comparing more specific age groups, such as young vs. middle-aged vs. older-aged adults. These findings also point out the need for cultural replications of this research, investigating how the FoMO scale performs in other non-western cultures (e.g., Korea, countries of the global south). Lastly, our lifespan sample was recruited with the snowball sampling technique and may not be representative.

To conclude, by providing a validated French version, this study supports cross-cultural and lifespan research on how digital technologies shape social and psychological well-being. Future research must replicate these results in middle and older aged individuals and investigate in more depth the mechanisms that link FoMO and other psychological processes. As our study, in line with prior work, demonstrates that FoMO has important implications for physical, mental and cognitive health and may, with rising technology and social media engagement, become more and more relevant for individuals across the lifespan, the need to better understand this phenomenon increases, putting it in the spotlight of discussions about information and communication technology interventions.

## Supplementary Information

Below is the link to the electronic supplementary material.

Supplementary Material 1 (DOCX 14.8 kb)

Supplementary Material 2 (DOCX 19.3 kb)

Supplementary figure 1 (PNG 545 kb)

Supplementary Material 3 (TIF 92.7 kb)

Supplementary Material 4 (DOCX 27.8 kb)

Supplementary Material 5 (DOCX 15.8 KB kb)

## Data Availability

Data is available on https://osf.io/fmbvt/overview (https://doi.org/10.17605/OSF.IO/FMBVT).

## Declarations

### Preprint

The preprint of this publication is available on https://doi.org/10.31219/osf.io/pg5yh.
