# Supplementary material for: Validation of the French fear of missing out (FoMO) scale across the lifespan
Source: Curr Psychol. 2026 Jan 17;45(3):217. doi: 10.1007/s12144-025-08669-z (PMC13652940; doi:10.1007/s12144-025-08669-z)
Supplement: Supplementary file 1 [file 12144_2025_8669_MOESM1_ESM.docx]

| **Appendix A** | |
| --- | --- |
| Table 1. Translation of the English FoMO scale in French | |
| **English** | **French** |
| 1. I fear others have more rewarding experiences than me | 1. Je crains que d'autres aient des expériences plus enrichissantes que moi. |
|  |  |
| 2. I fear my friends have more rewarding experiences than me | 2. Je crains que mes amis aient des expériences plus enrichissantes que moi. |
|  |  |
| 3. I get worried when I find out my friends are having fun without me | 3. Je m'inquiète lorsque je découvre que mes amis s'amusent sans moi. |
|  |  |
| 4. I get anxious when I don’t know what my friends are up to | 4. Je suis anxieux-se lorsque je ne sais pas ce que mes amis font. |
|  |  |
| 5. It is important that I understand my friends “in jokes” | 5. Il est important que je comprenne les "blagues" de mes amis. |
|  |  |
| 6. Sometimes, I wonder if I spend too much time keeping up with what is going on | 6. Parfois, je me demande si je ne passe pas trop de temps à me tenir au courant de ce qui se passe. |
|  |  |
| 7. It bothers me when I miss an opportunity to meet up with friends | 7. Cela me dérange lorsque je rate une occasion de rencontrer des amis. |
|  |  |
| 8. When I have a good time it is important for me to share the details online (e.g. updating status) | 8. Lorsque je passe un bon moment, il est important pour moi de partager les détails en ligne (par exemple, en mettant à jour mon statut). |
|  |  |
| 9. When I miss out on a planned get-together it bothers me | 9. Lorsque je rate une rencontre prévue, cela me dérange. |
|  |  |
| 10. When I go on vacation, I continue to keep tabs on what my friends are doing | 10. Lorsque je pars en vacances, je continue à suivre ce que font mes amis. |
|  |  |
