# Supplementary material for: Validation of the French fear of missing out (FoMO) scale across the lifespan
Source: Curr Psychol. 2026 Jan 17;45(3):217. doi: 10.1007/s12144-025-08669-z (PMC13652940; doi:10.1007/s12144-025-08669-z)
Supplement: Supplementary file 2 [file 12144_2025_8669_MOESM2_ESM.docx]

**Appendix B**

Table 1 *Means, standard deviations, and correlations with confidence intervals for variables in wave 2*

| Variable | *M* | *SD* | 1 | 2 | 3 | 4 | 5 | 6 | 7 | 8 | 9 |
| --- | --- | --- | --- | --- | --- | --- | --- | --- | --- | --- | --- |
|  |  |  |  |  |  |  |  |  |  |  |  |
| 1. w2other_more_expe_n | 1.96 | 1.09 |  |  |  |  |  |  |  |  |  |
|  |  |  |  |  |  |  |  |  |  |  |  |
| 2. w2friends_more_expe_n | 1.83 | 1.06 | .83** |  |  |  |  |  |  |  |  |
|  |  |  | [.80, .85] |  |  |  |  |  |  |  |  |
|  |  |  |  |  |  |  |  |  |  |  |  |
| 3. w2friends_fun_without_me_n | 1.73 | 1.03 | .52** | .59** |  |  |  |  |  |  |  |
|  |  |  | [.45, .58] | [.52, .64] |  |  |  |  |  |  |  |
|  |  |  |  |  |  |  |  |  |  |  |  |
| 4. w2anxious_friend_doing_n | 1.30 | 0.74 | .37** | .40** | .62** |  |  |  |  |  |  |
|  |  |  | [.29, .45] | [.32, .47] | [.56, .67] |  |  |  |  |  |  |
|  |  |  |  |  |  |  |  |  |  |  |  |
| 5. w2understand_friends_jokes_n | 2.49 | 1.18 | .27** | .29** | .41** | .34** |  |  |  |  |  |
|  |  |  | [.19, .36] | [.20, .37] | [.33, .49] | [.26, .42] |  |  |  |  |  |
|  |  |  |  |  |  |  |  |  |  |  |  |
| 6. w2miss_opportunity_n | 2.48 | 1.20 | .22** | .25** | .43** | .38** | .40** |  |  |  |  |
|  |  |  | [.13, .31] | [.17, .34] | [.35, .50] | [.29, .45] | [.32, .47] |  |  |  |  |
|  |  |  |  |  |  |  |  |  |  |  |  |
| 7. w2share_details_online_n | 1.46 | 0.88 | .19** | .19** | .33** | .35** | .16** | .18** |  |  |  |
|  |  |  | [.10, .27] | [.10, .28] | [.25, .41] | [.27, .43] | [.07, .25] | [.09, .27] |  |  |  |
|  |  |  |  |  |  |  |  |  |  |  |  |
| 8. w2bothers_miss_meet_n | 2.95 | 1.26 | .18** | .20** | .28** | .26** | .36** | .56** | .11* |  |  |
|  |  |  | [.09, .27] | [.11, .28] | [.20, .36] | [.17, .34] | [.28, .44] | [.50, .62] | [.02, .20] |  |  |
|  |  |  |  |  |  |  |  |  |  |  |  |
| 9. w2keep_info_vacation_n | 2.18 | 1.22 | .21** | .21** | .36** | .31** | .32** | .31** | .36** | .29** |  |
|  |  |  | [.12, .29] | [.12, .30] | [.28, .44] | [.23, .39] | [.23, .40] | [.23, .39] | [.28, .44] | [.20, .37] |  |
|  |  |  |  |  |  |  |  |  |  |  |  |
| 10. w2to_much_info | 1.87 | 1.12 | .39** | .40** | .40** | .44** | .32** | .26** | .32** | .19** | .35** |
|  |  |  | [.31, .46] | [.32, .47] | [.32, .47] | [.37, .51] | [.24, .40] | [.17, .34] | [.24, .40] | [.10, .28] | [.27, .43] |
|  |  |  |  |  |  |  |  |  |  |  |  |

*Note.* *M* and *SD* are used to represent mean and standard deviation, respectively. Values in square brackets indicate the 95% confidence interval for each correlation. The confidence interval is a plausible range of population correlations that could have caused the sample correlation (Cumming, 2014). * indicates *p* < .05. ** indicates *p* < .01.
