# Supplementary material for: Validation of the French fear of missing out (FoMO) scale across the lifespan
Source: Curr Psychol. 2026 Jan 17;45(3):217. doi: 10.1007/s12144-025-08669-z (PMC13652940; doi:10.1007/s12144-025-08669-z)
Supplement: Supplementary file 3 [file 12144_2025_8669_MOESM4_ESM.docx]

**Appendix D**

**Table B. Confirmatory Factor Analyses of FoMO Scale (one factor models)**

|  | **1-Factor (all items)** | **1-Factor (excluding item 1)** | **1-Factor (excluding items 1 & 6)** |
| --- | --- | --- | --- |
|  |  |  |  |
| I fear others have more rewarding experiences than me (Item 1) | .61 | - | - |
| I fear my friends have more rewarding experiences than me (Item 2) | .69 | .49 | .49 |
| I get worried when I find out my friends are having fun without me (Item 3) | .72 | .72 | .73 |
| I get anxious when I don’t know what my friends are up to (Item 4) | .53 | .59 | .60 |
| It is important that I understand my friends “in jokes” (Item 5) | .46 | .47 | .46 |
| Sometimes, I wonder if I spend too much time keeping up with what is going on (Item 6) | .40 | .42 | - |
| It bothers me when I miss an opportunity to meet up with friends (Item 7) | .48 | .57 | .55 |
| When I miss out on a planned get-together it bothers me (Item 8) | .31 | .38 | .37 |
| When I have a good time, it is important for me to share the details online (Item 9) | .22 | .29 | .27 |
| When I go on vacation, I continue to keep tabs on what my friends are doing (Item 10) | .40 | .49 | .47 |
| CFI | .56 | .75 | .72 |
| TLI | .43 | .66 | .61 |
| RMSEA | .19 | .13 | .15 |
| RMSEA p-value | <.001 | <.001 | <.001 |
| SRMR | .10 | .07 | .08 |
| Chi2 | 828.56 | 242.99 | 251.92 |
| Chi2 p-value | <.001 | <.001 | <.001 |
| Df | 35 | 27 | 20 |
| AIC | 13710.78 | 14054.78 | 12408.05 |
| BIC | 13826.06 | 14170.80 | 12511.18 |
| SABIC | 13740.36 | 14085.09 | 12434.99 |

**Table C. Confirmatory Factor Analyses of FoMO Scale (three factors models)**

|  | **3-Factors (all items)** | | | | **3-Factors (excluding item 1)** | | | | **3-Factors (excluding items 1 & 6)** | | |
| --- | --- | --- | --- | --- | --- | --- | --- | --- | --- | --- | --- |
|  | **Factor 1** | **Factor 2** | **Factor 3** | **Factor 1** | | **Factor 2** | **Factor 3** | **Factor 1** | | **Factor 2** | **Factor 3** |
| I fear others have more rewarding experiences than me (Item 1) | .80 |  |  | - | | - | - | - | | - | - |
| I fear my friends have more rewarding experiences than me (Item 2) | .98 |  |  | .54 | |  |  | .53 | |  |  |
| I get worried when I find out my friends are having fun without me (Item 3) | .50 |  |  | .88 | |  |  | .89 | |  |  |
| I get anxious when I don’t know what my friends are up to (Item 4) | .31 |  |  | .61 | |  |  | .61 | |  |  |
| It is important that I understand my friends “in jokes” (Item 5) |  | .46 |  |  | | .46 |  |  | | .46 |  |
| Sometimes, I wonder if I spend too much time keeping up with what is going on (Item 6) |  |  | .39 |  | |  | .42 | - | | - | - |
| It bothers me when I miss an opportunity to meet up with friends (Item 7) |  | .81 |  |  | | .60 |  |  | | .60 |  |
| When I miss out on a planned get-together it bothers me (Item 8) |  | .61 |  |  | | .83 |  |  | | .82 |  |
| When I have a good time, it is important for me to share the details online (Item 9) |  |  | .47 |  | |  | .47 |  | |  | .45 |
| When I go on vacation, I continue to keep tabs on what my friends are doing (Item 10) |  |  | .71 |  | |  | .69 |  | |  | .84 |
| CFI | .81 | | | | .95 | | | | .97 | | |
| TLI | .73 | | | | .93 | | | | .95 | | |
| RMSEA | .13 | | | | .06 | | | | .05 | | |
| RMSEA p-value | <.001 | | | | .19 | | | | .36 | | |
| SRMR | .10 | | | | .05 | | | | .04 | | |
| Chi2 | 280.21 | | | | 67.48 | | | | 42.67 | | |
| Chi2 p-value | <.001 | | | | <.001 | | | | <.001 | | |
| Df | 32 | | | | 24 | | | | 17 | | |
| AIC | 15307.78 | | | | 13873.37 | | | | 12207.15 | | |
| BIC | 15449.58 | | | | 14002.28 | | | | 12323.17 | | |
| SABIC | 15344.83 | | | | 13907.05 | | | | 12237.46 | | |

**Table D. Confirmatory Factor Analyses of FoMO Scale (three lower-level factors with one higher-level factor models)**

|  | **1 Higher-Order with 3 Lower-Order Factors (all items)** | | | | | **1 Higher-Order with 3 Lower-Order Factors (excluding item 1)** | | | **1 Higher-Order with 3 Lower-Order Factors (excluding items 1 & 6)** | | |
| --- | --- | --- | --- | --- | --- | --- | --- | --- | --- | --- | --- |
|  | **Factor 1** | | **Factor 2** | | **Factor 3** | **Factor 1** | **Factor 2** | **Factor 3** | **Factor 1** | **Factor 2** | **Factor 3** |
| I fear others have more rewarding experiences than me (Item 1) | .80 |  | |  | | - | - | - | - | - | - |
| I fear my friends have more rewarding experiences than me (Item 2) | .98 |  | |  | | .54 |  |  | .53 |  |  |
| I get worried when I find out my friends are having fun without me (Item 3) | .50 |  | |  | | .88 |  |  | .89 |  |  |
| I get anxious when I don’t know what my friends are up to (Item 4) | .31 |  | |  | | .61 |  |  | .61 |  |  |
| It is important that I understand my friends “in jokes” (Item 5) |  | .46 | |  | |  | .46 |  |  | .46 |  |
| Sometimes, I wonder if I spend too much time keeping up with what is going on (Item 6) |  |  | | .39 | |  |  | .42 | - | - | - |
| It bothers me when I miss an opportunity to meet up with friends (Item 7) |  | .61 | |  | |  | .83 |  |  | .60 |  |
| When I miss out on a planned get-together it bothers me (Item 8) |  | .81 | |  | |  | .60 |  |  | .82 |  |
| When I have a good time, it is important for me to share the details online (Item 9) |  |  | | .47 | |  |  | .47 |  |  | .45 |
| When I go on vacation, I continue to keep tabs on what my friends are doing (Item 10) |  |  | | .71 | |  |  | .69 |  |  | .84 |
| Superfactor loadings | .32 | .73 | | .77 | | .65 | .71 | .79 | .64 | .73 | .64 |
| CFI | .81 | | | | | .95 | | | .97 | | |
| TLI | .73 | | | | | .93 | | | .95 | | |
| RMSEA | .13 | | | | | .06 | | | .05 | | |
| RMSEA p-value | <.001 | | | | | .19 | | | .36 | | |
| SRMR | .10 | | | | | .05 | | | .04 | | |
| Chi2 | 280.21 | | | | | 67.48 | | | 42.67 | | |
| Chi2 p-value | <.001 | | | | | <.001 | | | <.001 | | |
| Df | 32 | | | | | 24 | | | 17 | | |
| AIC | 15307.78 | | | | | 13873.37 | | | 12207.15 | | |
| BIC | 15449.58 | | | | | 14002.28 | | | 12323.17 | | |
| SABIC | 15344.83 | | | | | 13907.05 | | | 12237.46 | | |
