# Supplementary material for: Validation of the French fear of missing out (FoMO) scale across the lifespan
Source: Curr Psychol. 2026 Jan 17;45(3):217. doi: 10.1007/s12144-025-08669-z (PMC13652940; doi:10.1007/s12144-025-08669-z)
Supplement: Supplementary file 4 [file 12144_2025_8669_MOESM5_ESM.docx]

**Appendix E**

| Table A. Measurement Invariance of the Model with Three Subfactors and One Superfactor, Comparing Young vs Older Aged Individuals (Item 10 Removed from all models) | | | | | | | | | | | | |
| --- | --- | --- | --- | --- | --- | --- | --- | --- | --- | --- | --- | --- |
|  | CFI | ΔCFI | TLI | RMSEA | RMSEA p-value | SRMR | Chi2 | Chi2 p-value | df | AIC | BIC | SABIC |
| Age (young vs old) |  |  |  |  |  |  |  |  |  |  |  |  |
| Configural Invariance | .965 | - | .939 | .057 | .321 | .046 | 44.63 | <0.01 | 24 | 9569.70 | 9763.58 | 9617.57 |
| Metric Invariance | .960 | .005 | .944 | .054 | .373 | .054 | 53.28 | 0.006 | 30 | 9567.54 | 9736.13 | 9609.16 |
| Scalar Invariance (achieved) | .948 | .012 | .933 | .059 | .254 | .060 | 62.50 | 0.001 | 33 | 9572.35 | 9728.29 | 9610.85 |
